# Genomic characterization of circulating human respiratory syncytial viruses A and B in Kuwait using whole-genome sequencing

**DOI:** 10.1128/spectrum.00159-24

**Published:** 2024-05-29

**Authors:** Nada Madi, Hussain A. Safar, Anfal Al-Adwani, Mohammed Sadeq, Mariam Al-Turab

**Affiliations:** 1 Virology Unit, Department of Microbiology, Faculty of Medicine, Kuwait University, Kuwait City, Kuwait; 2 Research Core Facility and OMICS Research Unit, Faculty of Medicine, Kuwait University, Kuwait City, Kuwait; 3 Jaber Al-Ahmad Armed Forces Hospital, Ministry of Health, Kuwait City, Kuwait; Johns Hopkins Medicine, Baltimore, Maryland, USA

**Keywords:** respiratory syncytial virus, whole-genome sequencing, nanopore sequencing, Kuwait

## Abstract

The human respiratory syncytial virus (RSV) is considered one of the most common viruses that infect children globally. The virus is known to have extensive gene sequence variability within and between RSV groups A and B globally; however, there is no information on the whole-genome characterization and diversity of RSV in Kuwait. Therefore, this study aimed to sequence the entire genome of RSV strains isolated from patients with acute respiratory tract infection (ARTI) in Kuwait. Therefore, this study aimed to sequence the entire genome of RSV strains isolated from patients with ARTI in Kuwait. Between January 2020 and September 2022, 7,093 respiratory samples were collected from hospitalized infants, children, and adults and were analyzed for respiratory viruses by multiplex real-time PCR. Whole-genome sequencing using the Oxford Nanopore sequencing technology was performed on 84 RSV-positive samples. The results revealed a higher prevalence of group A (76%) than group B (24%) RSV isolates. Phylogenetic analysis showed that RSV-A strains clustered with the GA2.3.5 sub-genotype and RSV-B strains clustered with the GB5.0.5a sub-genotype; however, forming new lineages of RSV-A and RSV-B circulated in Kuwait during this period. Genetic variability was higher among the group A viruses than group B viruses, and the rate of synonymous and missense mutations was high in genes other than the G protein-coding gene. We also detected several known and unique molecular markers in different protein-coding genes. This is the first study in Kuwait to characterize the whole genomes of RSV A and B to identify the circulating genotypes, comprehend the genetic diversity and the evolution of the virus, and identify important genetic markers associated with specific genotypes.

**IMPORTANCE:**

Whole-genome sequencing of respiratory syncytial virus (RSV) strains in Kuwait using MinION Nanopore technology was used to characterize and analyze the genotypes and sub-genotypes of the RSV circulating among patients with acute respiratory tract infections in Kuwait. This study also identified known and unknown gene mutations and imported genetic markers associated with specific genotypes. These results will assist in establishing a framework for RSV classification and allow for a better consideration of the mechanisms leading to the generation of diversity of RSV. In addition, these data will allow a comparison of vaccine viruses with those in Kuwait, providing useful insights into future vaccine and therapy strategies for RSV in Kuwait.

## INTRODUCTION

The human respiratory syncytial virus (HRSV) is among the most significant pathogen-causing infections in infants and children and is associated with considerable morbidity and mortality worldwide (1). It was estimated that 3.4 million children under 5 years of age are hospitalized each year with severe respiratory syncytial virus (RSV) lower respiratory tract infection, with the peak incidence in children younger than 6 months of age. Moreover, up to 200,000 deaths occur annually in developing countries, with most deaths occurring in children under 1 year of age (2, 3). RSV can also infect adults in the elderly, resulting in severe illness (4, 5). The clinical manifestations of RSV range from mild upper respiratory tract infection and otitis media to severe and possibly life-threatening lower respiratory tract infection (LRTI). In infants infected with RSV, bronchiolitis is the most common and serious form of LRTI. Nevertheless, pneumonia and croup were also recorded (6, 7).

RSV has a non-segmented negative-stranded RNA genome of around 15,200 nucleotides in size, consisting of 10 genes encoding 11 proteins (8). There are two antigenic groups of RSV, A and B, and each group is classified into many distinct subgroups according to antigenic and genomic sequence differences in G glycoprotein (9, 10). There are 22 genotypes of RSV-A, GA1–7, SAA1–2, NA1–4, ON1–4, CB-A, TN1–2, and LBA1–2, while there are 36 RSA-B genotypes, GB1–13, THB, BA1–14, BA-CCA, BA-CCA, SAB1–4, and URU1–2 (11, 12). Because the G-protein has large genetic variations, this leads to the emergence of new variants.

RSV genotypes circulate worldwide, with the same predominant clades of viruses being found in countries worldwide (13–15). Molecular analyses revealed that several intra-group viral genotypes or clades are present concurrently in any season and area; however, even in adjacent cities, the circulating strains may vary, resulting in a wide-ranging set of circulating viruses that can adapt to herd immunity (16, 17). It was noticed that both RSV genotypes and distinct clades can co-circulate locally in successive years; however, they alternate predominance in 1- or 2-year cycles (18).

In Kuwait, among 305 respiratory samples collected between January and mid-December 2016 from patients with acute respiratory tract infection (ARTI) in various hospitals in Kuwait, 77 (25.2%) were positive for RSV. In addition, group A viruses were predominant over group B viruses; the RSV-A group was detected in 52 (67.5%) positive samples, while the RSV-B group was detected in 25 (32.5%) positive samples. Phylogenetic analysis showed that all RSV-A strains were grouped into eight identical sequences of untyped strains. On the other hand, 12 RSV-B strains belonged to the RSV-B/BA10 genotype, while the rest were untyped (19).

Several studies have shown that RSV group A is associated with the severe form of the disease compared with group B (1, 20, 21). Contrariwise, one study reported a higher severity in group B compared with group A infection (22). Preventing the diseases formed by RSV is possible soon as several clinical trials develop an effective RSV vaccine. Many clinical trials with live-attenuated virus vaccines against RSV have been reported; some are near completion (23). As a result of the emergence of novel RSV genotypes, a potential association between the RSV genotype and disease severity or geographic and time-based restriction of virus circulation has been reported in many studies (1, 24). Also, it has been shown that RSV genetic diversity is an important factor that allows for reinfections to occur, which should be considered in vaccine development (24). Consequently, an efficient genotyping system is needed to reflect RSV genetic diversity. Earlier RSV sequencing largely relied on complete or partial G gene sequencing for diagnosis genotyping. However, other genes proved to be involved in the evolution of RSV that should also be considered.

Whole-genome sequencing of viral strains is the ideal solution, especially in cases where genotyping virus based on partial sequencing could be more productive. Most RSV sequences in GeneBank are partial-coding sequences with a few whole-genome sequences. Consequently, whole‐genome analyses will deliver a more inclusive consideration of the evolutionary processes of RSV; also, it will identify the variable and conserved regions of the virus for the development of diagnostic tools and vaccines. For strain classification, all the attention was paid to the G gene, which shows the highest variability, and not so much attention has been paid to other viral genes. Nonetheless, other genes also show substantial variation that could help genotype classification. Variations in genes other than the G gene may influence strain virulence and circulation patterns of the virus through time (25).

In Kuwait, studies on whole-genome sequences of RSV are lacking; therefore, whole-genome sequencing of RSV using MinION Nanopore technology was used to identify and analyze the precise genotypes and sub-genotypes of the RSV circulating among patients with acute respiratory tract infections in Kuwait. This study also identified single-nucleoid polymorphisms (SNP) and imported genetic markers associated with specific genotypes. These results will assist in establishing a framework for RSV classification and allow for a better consideration of the mechanisms leading to the generation of RSV diversity.

## RESULTS

### Demographics of the study population

**Fig 1:**
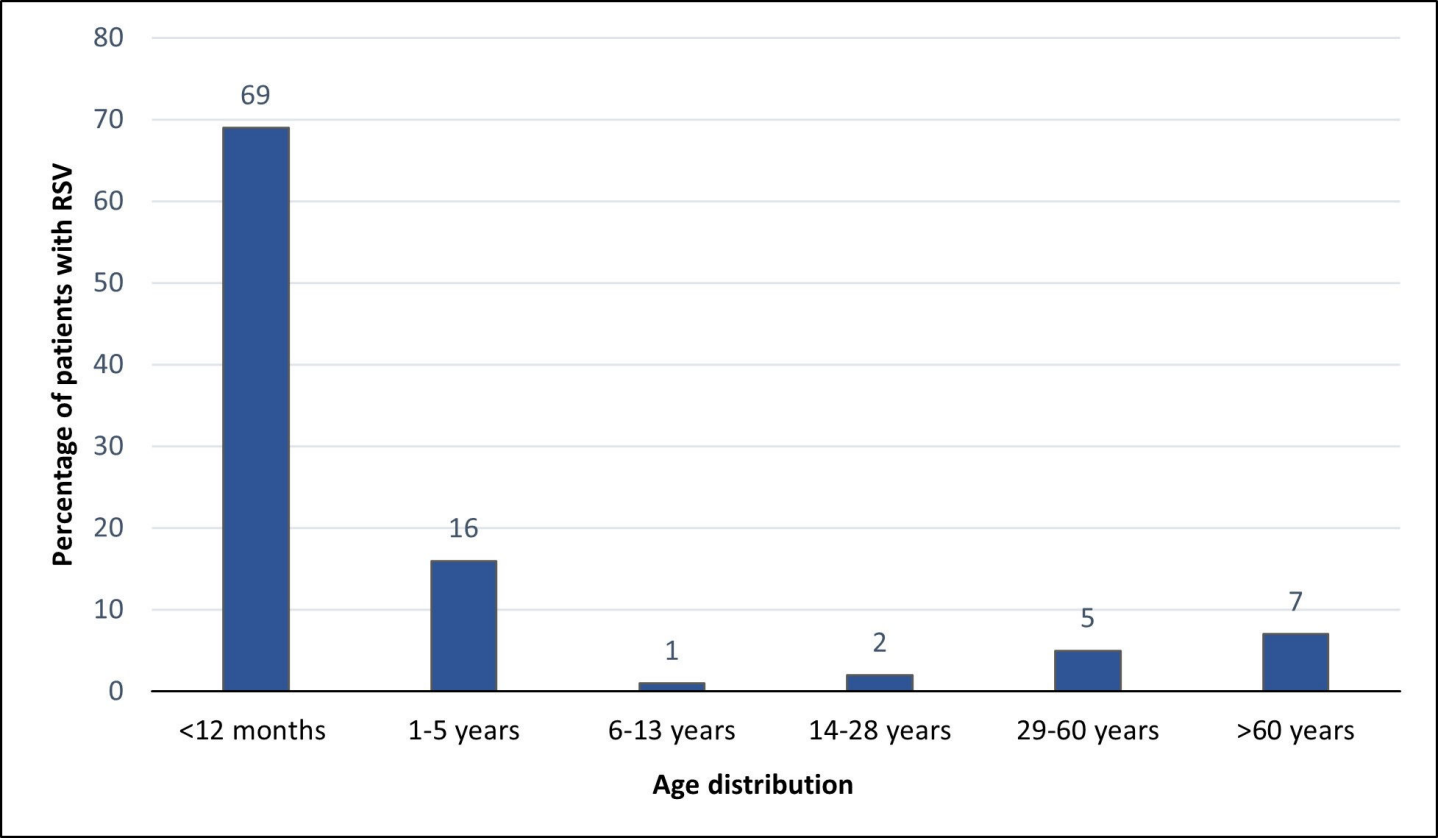
Age distribution of RSV-positive patients in Kuwait (%) (*n* = 490).

From January 2020 to September 2022, 7,093 respiratory samples were collected from hospitalized infants, children, and adults and analyzed for respiratory viruses by multiplex real-time PCR, resulting in 490 (6.9%) RSV-positive samples. Of all RSV-positive patients, 245 (50%) were females, and 245 (50%) were males; their ages ranged from less than 1 year to 97 years (median: less than 1 year old). Figure 1 shows the occurrence of RSV infection in the various age groups. The majority (69%, *n* = 338) of the patients were younger than 12 months, 16% (*n* = 79) of patients was between 1 and 5 years old, 7% (*n* = 34) of patients was over 60 years old, 5% (*n* = 27) of patients was 29–60 years old, 2% (*n* = 8) of patients was 14–28 years old, and only 1% (*n* = 4) of patients was 6–13 years old. As for the seasonal RSV distribution among Kuwaiti patients between January 2020 and September 2022, Fig. 2 shows that the peak of positivity was detected in October 2021.

**Fig 2:**
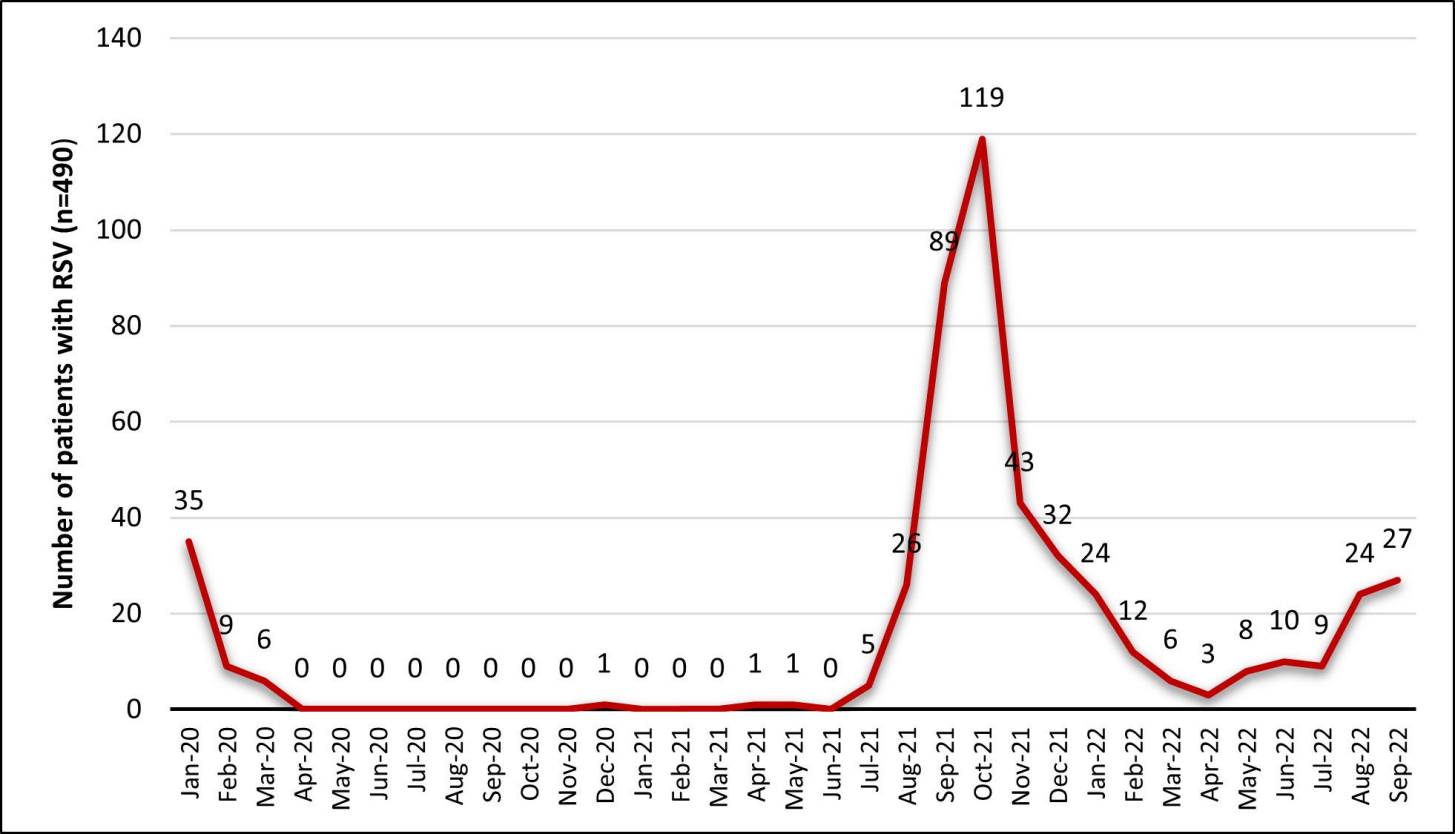
Seasonal trends of RSV in Kuwait from January 2020 to September 2022.

### Sequence alignment and assignment of RSV genotypes in Kuwait

**Fig 3:**
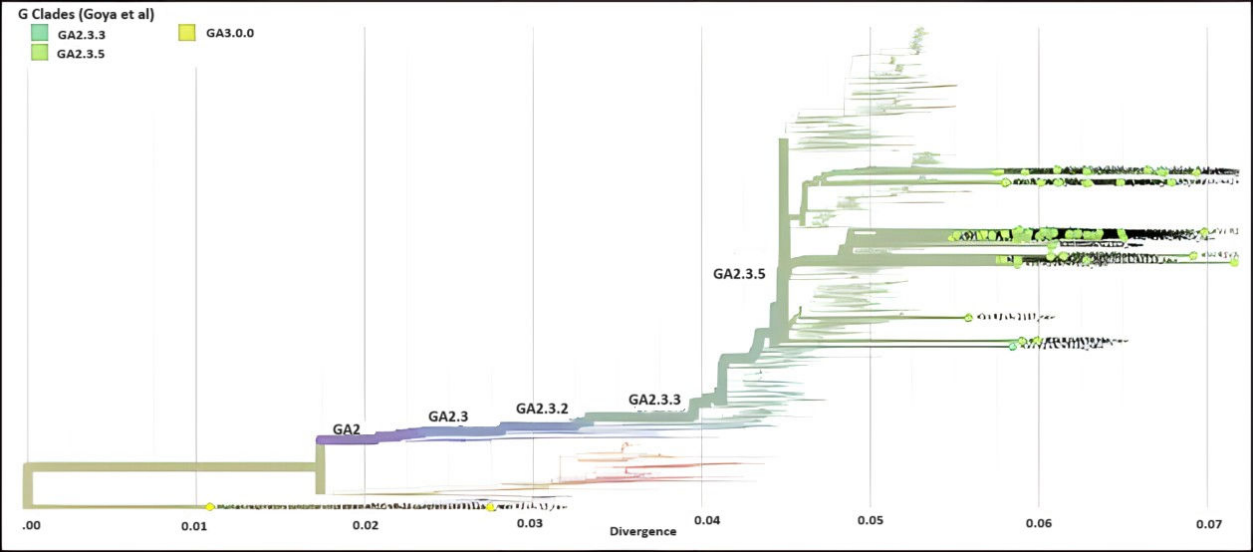
RSV-A genotype (*n* = 64) assignment according to Nextstrain classification from 2020 to 2022. Different colors denote different genotypes and sub-genotypes of RSV-A. Kuwaiti strains are green dots.

**Fig 4:**
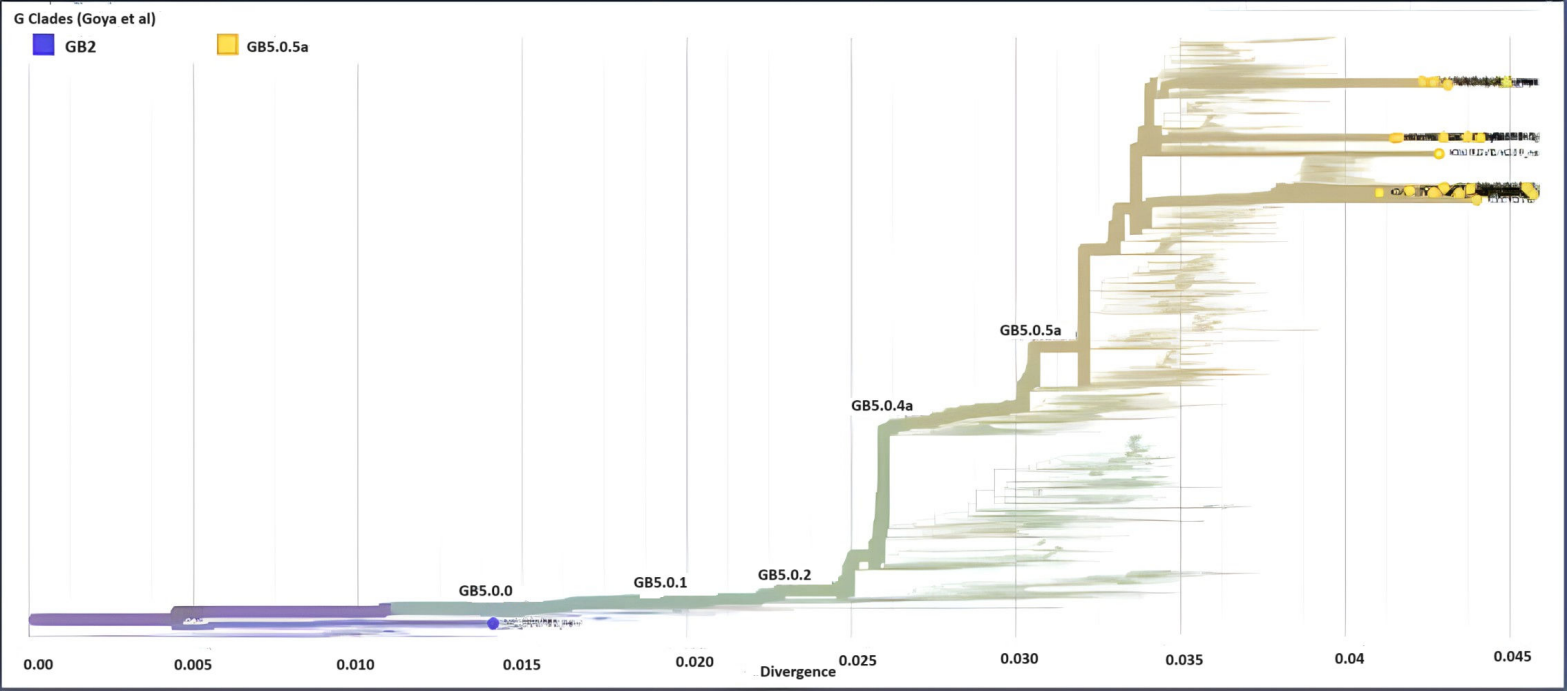
RSV-B genotype (*n* = 20) assignment according to Nextstrain classification from 2020 to 2022. Different colors denote different genotypes and sub-genotypes of RSV-B. Kuwaiti strains are in orange dots.

Between January 2020 and September 2022, 490 RSV-positive respiratory samples from patients in Kuwait were involved in this study. Out of these samples, we attained 84 high-quality sequences of RSV isolate for downstream analysis. Using the 15,222-nt prototype of RSV-A (AY911262) as a reference genome and 15,226-nt RSV-B reference genome (NC_001781), we have successfully assembled 84 RSV consensus genomes and were used for phylogenetic analysis. Sequence analysis revealed that out of 84 RSV genomes, 64 (76%) were from the RSV-A genotype, while 20 (24%) were from the RSV-B genotype. Initial alignment of the sequences using Nextstrain analysis revealed that all RSV-A sequences were assigned into three main G clades: GA2.3.5 (*n* = 62; 97%), GA2.3.3 (*n* = 1; 1.5%), and GA3 (*n* = 1, 1.5%); however, all RSV-B sequences were from the GB5.0.5a sub-genotype (Fig. 3 and 4).

### Phylogenetic analysis of circulating RSV-A and RSV-B in Kuwait

From the 84 RSV-A and RSV-B genomes and reference sequences, phylogenetic analysis was constructed using the maximum likelihood method and Tamura-Nei/JTT matrix-based models of MEGA11. The percentage of nucleotide identity between the Kuwaiti RSV-A strains was 92%, while the rate of nucleotide identity between Kuwaiti RSV-B strains was 97%. The evolutionary divergence between sequences of RSV-A measured by pairwise distance (*p*-distance) showed an overall average pairwise distance of 0.015, and the highest intergenotypic distance (*P* = 0.038) was detected between KW/RSVA/15-KW/RSVA/53109 isolates, while the lowest *P*-distance (*P* = 0.000685) was between KW/RSVA/0283-KW/RSVA/45178 and between KW/RSVA/44521-KW/RSVA/36 isolates. On the other hand, the evolutionary divergence between sequences of RSV-B measured by pairwise distance showed an overall average pairwise distance of 0.021 and the highest intergenotypic distance (*P* = 0.0228) was detected between KW/RSVB/3244-KW/RSVB/9761 isolates, while the lowest intergenotypic distance (*P* = 0.00265) was between KW/RSVB/10884-KW/RSVB/10505 isolates.

**Fig 5:**
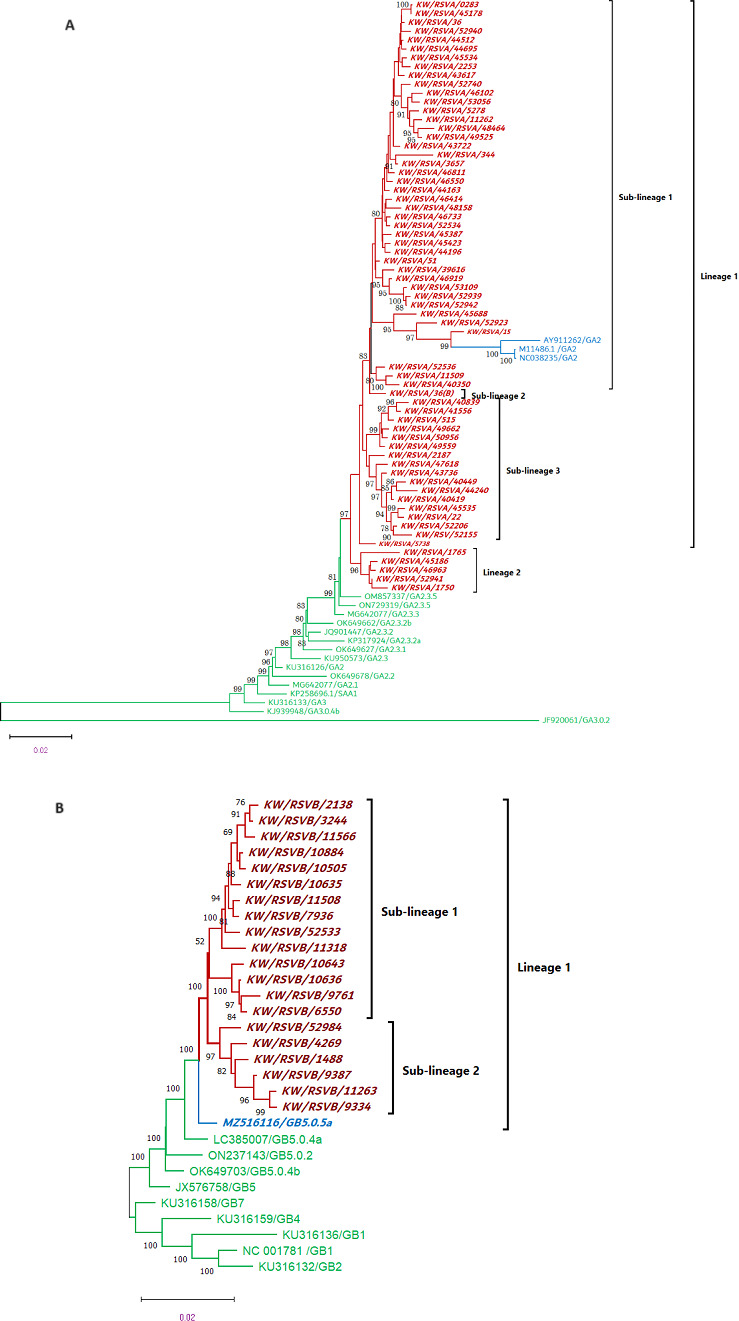
Phylogenetic analysis of RSV-A strain (**A**) and RSV-B (**B**) circulating in Kuwait, January 2020–September 2022. Whole-genome RSV-A and RSV-B sequences from Kuwaiti and reference strain sequences were used to generate a phylogenetic tree using the maximum likelihood method and general time reversible/JTT matrix-based models. Kuwaiti strains are in bold. The scale bar indicates the proportion of nucleotide substitutions, and the numbers at the branches are bootstrap values determined for 1,000 repetitions.

The phylogenetic tree (Fig. 5A) showed an overall *P* = 0.036 between RSV-A sequences and RSV-A reference strains. The 64 RSV-A sequences were grouped into two major lineages clustered with reference OM857337/GA2.3.5 with a low bootstrap support of 32% and pairwise distance of 0.03. Lineage 1 consisted of 59 RSV-A sequences closely related to lineage 2, which consisted of five sequences with a bootstrap support of 97%. Furthermore, the average *P-*distance between sequences in lineage 1 was 0.027, showing three separate sub-lineages. Within sub-lineage 1, KW/RSV-A/15 was closely related to GA2 reference strains (M11486.1, AY911262, and NC038235) with a bootstrap support of 99%. Sub-lineage 2 consisted of only one sequence closely related to sub-lineage 2 with a bootstrap support of 83% and base composition bias difference between sequences of 0.0126. Sub-lineage 3 sequences were related to sub-lineages 2 and 3 with a weak bootstrap support of 35%; however, the base composition bias difference between the sequences in this sub-lineage was 0.0149. The second lineage consisted of five RSV-A sequences with a strong bootstrap support of 96% and an average *P*-distance within this lineage of 0.0098 and base composition bias difference between the sequences of 0.0059 (Fig. 5A).

The evolutionary divergence between sequences of RSV-B showed an overall average pairwise distance of 0.014, and the highest intergenotypic distance (*P* = 0.0229) was detected between KW/RSVB/3244-KW/RSVB/9761 strains, while the lowest intergenotypic distance (*P* = 0.0026) was between KW/RSVB/10884-KW/RSVB/10505. The phylogenetic tree in Fig. 5B showed an overall *P* = 0.022 between RSV-B sequences and RSV-B reference strains. The 20 RSV-B sequences were grouped into one major lineage clustered with reference GB5.0.5a with a bootstrap support of 100%. This lineage was sub-divided into two sub-lineages with a bootstrap support of 100%. Sub-lineage 1 sequences were related to the base composition bias difference between sequences of 0.0116. Sub-lineage 2 sequences were closely linked with base composition bias difference between sequences of 0.0058.

### Time-scaled phylogeny of RSV-A and RSV-B from Kuwait and the world

**Fig 6:**
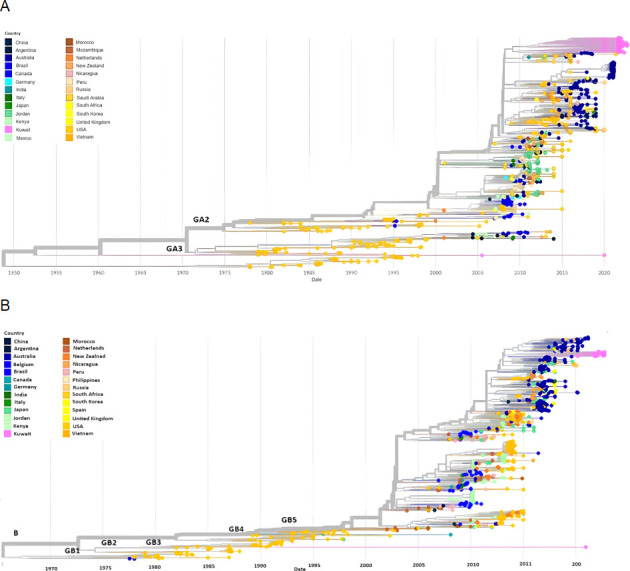
Time-scaled phylogenetic tree of RSV. (A) A tree representing 960 high-coverage genomes of RSV-A from 27 countries sampled between December 1977 and September 2022, including 64 RSV-A isolates from Kuwait. (B) A tree representing 888 high-coverage genomes of RSV-B from 27 countries sampled between July 1977 and September 2022, including 20 RSV-B isolates from Kuwait. The phylogeny was estimated using IQTree under the GTR substitution model and visualized with auspice. The tree was rooted with the RSV-A reference strain AY911262 and RSV-B reference strain NC_001781.

A time-scaled phylogenetic tree was constructed from the genome of 64 RSV-A strains in Kuwait and 960 high-coverage genomes sampled between December 1977 and September 2022 from 27 countries retrieved from the GenBank (Fig. 6A). Another tree was constructed from the genome of 20 RSV-B strains in Kuwait, and 888 high-coverage genomes were sampled between July 1977 and September 2022 from 27 countries, which were also retrieved from the GenBank (Fig. 6B). The temporal data showed that the earliest common ancestor of the Kuwaiti strains of RSV-A (genotype GA2; sub-genotype GA2.3.5) was introduced in the year 2011 with a divergence of 0.051, while the latest ancestor was introduced in the year 2020 with a divergence of 0.061 (Fig. 6A). The earliest common ancestor of the Kuwaiti strains of RSV-B (genotype GB5, sub-genotype GB5.0.5a) was introduced in the year 2014 with a divergence of 0.036, and the latest ancestor was introduced in the year 2021 with divergence of 0.048 (Fig. 6B).

### Association of RSV genomes from Kuwait with genomes from other countries

**Fig 7:**
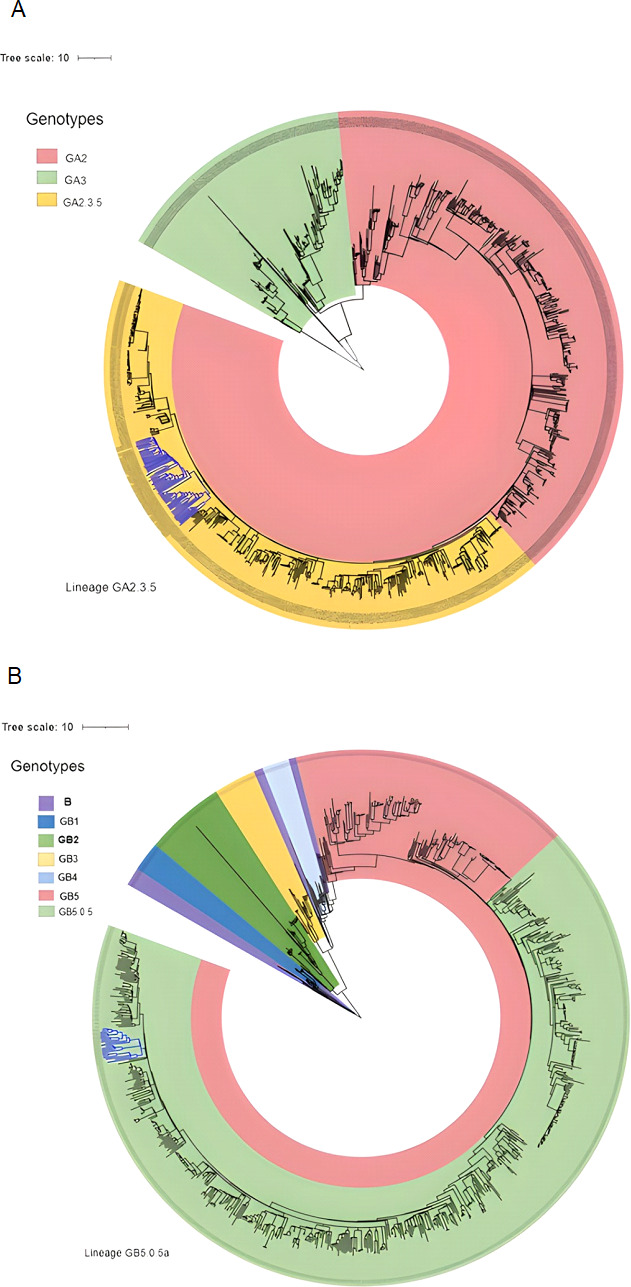
Maximum likelihood IQ TREE of 960 RSV-A (**A**) and 888 RSV-B (**B**) genomes from different countries retrieved from the GenBank and the Kuwaiti strains. Kuwaiti strains are in blue branches.

The viral phylogeny of 960 RSV-A and 888 RSV-B genomes from different countries and Kuwait is shown in Fig. 7A and B. The Kuwaiti RSV-A genotype GA2 (sub-genotype GA2.3.5) constituted 97% of the sampled genomes from Kuwait, showing a separate lineage of related Kuwaiti strains with a 97% bootstrap value. However, the Kuwaiti lineage clustered with genomes from Australia, India, Jordan, New Zealand, Saudi Arabia, and the United States, with a divergence of 0.48 from the common ancestor A genotype (Fig. 7A). On the other hand, all (100%) Kuwaiti RSV-B genotype GB (sub-genotype GB5.0.5a) showed a separate lineage. This Kuwaiti lineage clustered with a lineage that consisted of genomes from Australia with a divergence of 0.034 from the common ancestor B genotype (Fig. 7B).

### Individual gene analysis

SNP calling was performed in the genes of the Kuwaiti isolates’ RSV-A and RSV-B sequences using the maximum likelihood method under the most suitable model for each gene data. Numerous SNPs were found in their genomes, indicating high genetic diversity levels of RSV-A and RSV-B; the total number of SNPs in RSV-A sequences was larger than that in RSV-B sequences (RSV-A = 1359, RSV-B = 749). Among the 10 genes of the virus, the highest rate of coding SNP was detected in the G gene in both RSV-A and RSV-B, 15% and 11%, respectively (Table 1), while for the other genes, the SNP occurrence rates were lower, ranging from 6% in NS1, NS2, P, and M genes to 12% in the M2-2 gene for RSV-A and from 2% in the SH gene to 5% in the NS2 gene for RSV-B.

**TABLE 1:** Number of SNPs and rations of dN/dS for each gene of the RSV-A and RSV-B genomes^*a*^

Gene	Group	Length (bp)	No. of SNP	Percentage of SNP	Percentage of synonymous mutations	Percentage of missense mutations	dN/dS	Selection type
Non-structural protein 1 (NS1)	A	419	25	6	88	12	−0.030	Negative
	B	532	17	3	59	41	−0.0075	Negative
Non-structural protein 2 (NS2)	A	374	31	8	81	19	−0.043	Negative
	B	504	25	5	84	16	−0.032	Negative
Nucleoprotein (N)	A	1175	73	6	**96**	4	−0.036	Negative
	B	1202	43	4	93	7	−0.022	Negative
Phosphoprotein (P)	A	725	43	6	86	14	−0.025	Negative
	B	913	32	4	**94**	6	−0.038	Negative
Matrix protein (M)	A	770	43	6	93	7	−0.063	Negative
	B	954	34	4	91	9	−0.047	Negative
Small hydrophobic protein (SH)	A	194	11	6	73	27	−0.066	Negative
	B	412	8	2	25	**75**	0.0014	Negative
Attachment protein (G)	A	896	134	**15**	49	51	−0.032	Negative
	B	925	102	**11**	54	46	−0.037	Negative
Fusion protein (F)	A	1724	145	8	82	18	0.034	Negative
	B	1899	70	4	81	19	−0.025	Negative
M2-1 protein	A	584	48	8	88	12	−0.034	Negative
	B	588	21	4	81	19	−0.0137	Negative
M2-2 protein	A	272	32	12	44	**56**	0.003	Negative
	B	272	10	4	50	50	−0.0136	Negative
Large polymerase protein (L)	A	6497	506	8	84	16	−0.027	Negative
	B	6579	245	4	84	16	−0.020	Negative

^*a*^ Numbers in bold are the highest values.

Mutation analysis was conducted on RSV-A and RSV-B sequences, including all possible mutations: missense, synonymous, insertion, and deletion (Table 1). The data from our study found that, in general, RSV-A sequences had a greater number of synonymous and missense mutations (488,871, respectively) than the sequences of RSV-B, which had 411 synonymous mutations and 338 missense mutations. No insertion or deletion mutations were detected in the coding regions of RSV-A and RSV-B sequences; however, an insertion in the intergenic region (nt = 4,209) was detected in all the RSV-A sequences. Moreover, individual analysis of mutations in each gene revealed that the highest rate of missense mutations (75%) was in the SH protein gene of RSV-B genotypes, while the most increased missense mutations in RSV-A were in the M2-2 protein gene (56%). The lowest rate of missense mutations was detected in the N gene of RSV-A and the P gene in RSV-B (4% and 6%, respectively). The highest rate of synonymous mutations (96%) was detected in the N gene of RSV-A, while the highest rate of synonymous mutations in RSV-B was seen in the P protein gene (94%). The lowest rate of synonymous mutations in RSV-A (44%) was detected in the M2-2 gene, while the lowest rate of synonymous mutations (25%) in RSV-B was seen in the SH gene. The study also determined the dN-to-dS ratio for each gene of both RSV-A and RSV-B. The ratios for all the RSV-A and -B genes were <1, indicating a negative selection pressure in these genes (Table 1).

### Detection of molecular markers

**Fig 8:**
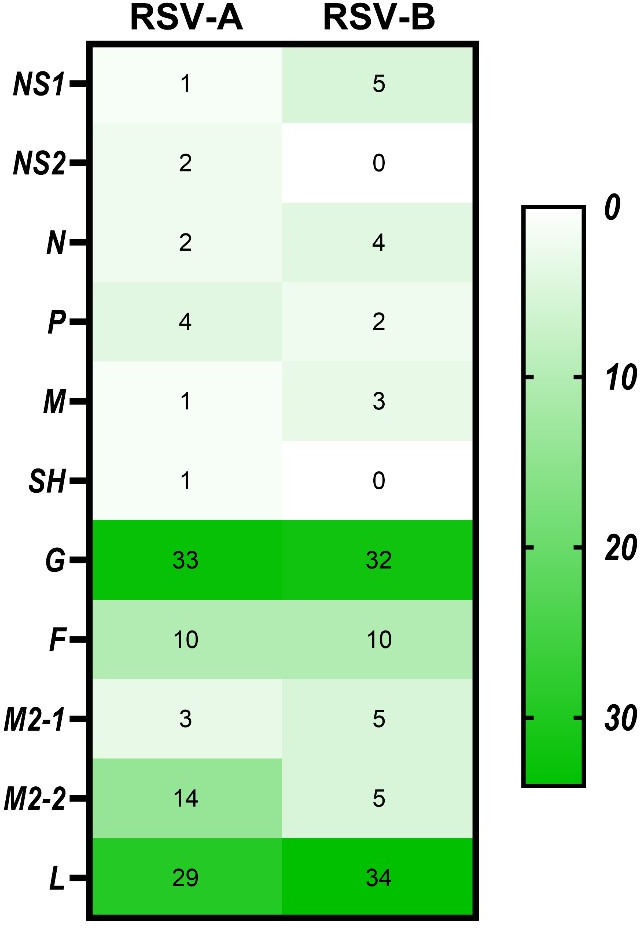
Heatmap demonstrates the percentage of molecular markers in each gene among RSV-A and RSV-B genotypes detected in Kuwait. The color scale indicates the significance of the correlation, with blue and white colors indicating the highest and lowest correlation, respectively.

Nucleotide sequences for each of the 10 RSV-A genes (NS1, NS2, N, P, M, SH, G, F, M2, and L), which are crossing from 3′UTR to 5′UTR were aligned and compared with the RSV-A reference sequence (Accession Number AY911262.1). In contrast, RSV-B genotype sequences were aligned and compared with the RSV-B reference sequence (accession number NC_001781.), and every variant at every site was recorded. The fixation percentage of changes in every amino acid position for each genotype was calculated. It was considered a molecular marker if the fixation percentage exceeded 75%. In total, 107 amino acid variants in the proteins of the RSV-A genotype fulfilled the criteria to be considered molecular markers (Table S1). From all amino acid molecular markers, 36 (34%) were in the G protein, 31 (29%) in the L protein, 15 (13%) in the M2-2 protein, 11 (10%) in the F protein, 4 (4%) in the *P* protein, 2 (2%) in both N and NS2, and 1 (1%) in the NS1, M, and SH proteins (Fig. 8). On the other hand, 73 amino acid variants in the total proteins of the RSV-B genotype were molecular markers (Table S2). Of all amino acid molecular markers, 25 (34%) were in the L protein, 23 (32%) in the G protein, 7 (10%) in the F protein, 4 (5%) in the NS1, M2-1, and M2-2 proteins, 3 (4%) in the N protein, and 1 (2%) in the P protein. At the same time, no molecular markers existed in NS2 and SH proteins.

## DISCUSSION

This study is the first in Kuwait to describe RSV variability among patients suffering from different respiratory symptoms between January 2020 and September 2022. RSV is a pathogen that infects the respiratory tracts of infants, children, and the elderly (26, 27). The virus is a large family containing two different antigenic groups further divided into distinct genotypes and sub-genotypes. Over the last few years, many RSV-A and RSV-B genotypes have been reported in the literature, where the diversity was evident within the G gene; therefore, the genotyping assignments were based on the analysis of the hypervariable region of the G gene only. As a result, when analyzed together, RSV strains might group in the same cluster based on the whole-genome sequence (28, 29). It is worth mentioning that more sequence variation was detected in the F and selected regions of the L gene. This study analyzed the entire RSV-A and RSV-B genome sequences for accurate classification. RSV was detected in 6.9% of the patients with respiratory illnesses in this study between 2020 and 2022, which was lower than what was recorded previously (25.2%) in Kuwait during the year 2016 (30) but slightly higher than the RSV incidence reported in Italy (31). However, the incidence of RSV is higher elsewhere in developing and emerging countries, where it can reach 18% (32). Of note, the lower incidence of RSV in this study is probably due to the severe acute respiratory syndrome coronavirus 2 (SARS-CoV-2) pandemic, which suppressed the circulation of RSV and other respiratory viruses. This study showed that RSV infection is significant in two main groups: children <5 years old and elderly ≥60 years old, which is compatible with data recorded earlier (31, 33, 34). Furthermore, the results showed that RSV-positive cases were mostly observed in the beginning and during the winter months (September to February) while declining in the spring and summer months. Such seasonal patterns were also consistent with other reports (35–37).

Sequence analysis of 84 RSV strains detected in Kuwait showed that the majority belonged to the RSV-A genotype (76%), while the rest belonged to the RSV-B genotype (24%). These results agree with others that showed the predominance of RSV-A over RSV-B genotypes in different populations (35, 38, 39). According to the Nextstrain classification, the GA2.3.5 sub-genotype was predominant in Kuwait, accounting for 97%, while only 1% of the strains belonged to sub0henotype GA2.3.3 and 1% belonged to sub-genotype GA3. On the other hand, the predominant RSV-B sub-genotype was the GB5.0.5a in Kuwait, where all the strains belong to this sub-genotype. In parallel to our results, Goya et al*.* (40) demonstrated the circulation of GA2.3.5, GA2.3.6a, and GA2.3.6b genetic lineages of RSV-A and GB5 genotype and GB5.0 sub-genotype with multiple genetic lineages GB5.0.2 and GB5.0.4a and the divergent GB5.0.5a and GB5.0.4c of RSV-B in Buenos Aires. Similarly, India reported the predominance of the GA2 genotype and the circulation of GA2.3.0, GA2, GA3.0.3, GA2.3.3, GA2.3.1, and GA2.3.5 sub-genotypes for group A RSV, in addition to the predominance of the GB5 genotype and the circulation of GB2, GB5.0.2, GB5.0.5c, and GB5.0.2 sub-genotypes for group B RSV (39, 41, 42). Data show that GA2 and GA3 genotypes in group A and GB5 and GB7 genotypes in group B are the most frequently detected genotypes globally (43). Our previous study in Kuwait demonstrated that many RSV-A strains were untyped based on G region analysis; however, few strains were genetically closer to ON1 divergent lineages, i.e., GA2.3.6a and GA2.3.6b. The study also showed that many RSV-B strains belonged to BA10, which includes divergent GB5.0.5a and GB5.0.4c.

Phylogenetic analysis revealed that the level of genetic variability was higher among the group A viruses (8%) than among group B viruses (3%). In addition, the analysis showed that the RSV-A strains were closely related with an overall intergenotype average *P*-distance of 0.021; RSV-B strains were closely related with an overall intergenotype average *P*-distance of 0.015. The 64 RSV-A sequences were grouped into two main lineages clustered with the GA2.3.5 reference strain but with a weak genetic relationship (bootstrap value = 32%) with a pairwise distance of 0.03 and a divergence of 9% between the sequences and GA2.3.5 reference strain. These results suggest the presence of unique new lineages of the GA2 clade of the RSV-A group in Kuwait, according to the RSV classification proposed by Goya et al*.* (43). Furthermore, lineage 1 (59 sequences) and lineage 2 (5 sequences) were closely related, with a strong bootstrap support of 97%. However, within lineage 1, three sub-lineages showed a close relationship (bootstrap of 80%) and a close relationship between some strains in sub-lineage 1 and the GA2 genotype reference strains. Furthermore, our results showed that the 20 RSV-B sequences were closely related (average *P* = 0.014) and clustered with GB5.0.5a reference strain (bootstrap support of 100%). RSV-B sequences were further subdivided into two sub-lineages, where the nucleotide difference between sequences in sub-lineage 1 was slightly higher than the nucleotide differences between sequences in sub-lineage 2 (base composition bias difference rates of 0.0116 and 0.0058, respectively).

To demonstrate the evolution and geographic distribution of RSV-A and RSV-B strains in Kuwait between 2020 and 2022 and other RSV-A and RSV-B strains from different countries, a time-scale phylogenetic tree was constructed and showed that the RSV-A genotype GA2 (GA2.3.5 sub-genotype) strains that circulated between 2020 and 2022 in Kuwait were introduced early in the year 2011, while RSV-B genotype GB5 (GB5.0.5a sub-genotype) strains that circulated in Kuwait were introduced in the year 2011. Notably, the first detection of the GA2 genotype of RSV-A was in 1974, and the first detection of RSV-B genotype GB5 was in 1996. In addition, the Kuwaiti RSV-A genomes clustered with genomes from other countries such as Australia, India, Jordan, New Zealand, Saudi Arabia, and the United States, while the Kuwaiti RSV-B genomes clustered with the genomes of Australia.

Like other RNA viruses, RSV is characterized by gene mutations, an important factor in the evolution of viruses. Mutation is a mechanism that enables the virus to adapt itself to a changed environment, such as the host’s immune pressure. This study showed high genetic diversity levels of RSV-A and RSV-B according to the number of SNP. Also, a comparison of mutation rates of different genes of RSV showed that the SNP rate in the G gene in both RSV-A and B was slightly higher than that of other genes; however, no positive selection was found in the G gene or other genes. This phenomenon suggests that the G gene and other genes exhibit genetic diversity that reflects the impact of host immune responses in the epidemic cycle. Our study showed that the number of synonymous and missense mutations was higher in RSV-A than in RSV-B, which may be credited to a wider prevalence of RSV-A. Surprisingly, our study showed that the highest rate of missense mutations among RSV-A genotypes was in the M2-2 gene. In contrast, the SH gene had the highest rate of missense mutations among RSV-B genotypes. M2-2 and SH proteins are important viral proteins; the second open reading frame (ORF) of the viral gene M2 expresses the M2-2 protein. During infection, M2-2 acts as the polymerase cofactor that promotes genome replication (44). On the other hand, the SH protein is a small hydrophobic protein that may have a role in viral fusion or in changing membrane permeability (45). Also, the results showed a high rate of synonymous mutations in the N gene of RSV-A and a high rate of synonymous mutations in the P gene of RSV-B. Therefore, these results suggest that the increased diversity in G protein is not the main contributor to the evolution of RSV. Regarding the molecular markers, the results showed more molecular markers in RSV-A than in RSV-B strains. The highest rate of molecular markers (34%) was detected in the G protein of RSV-A, while the L protein came second. On the other hand, the highest rate (34%) of molecular markers was in the L protein of RSV-B genotypes, followed by the G protein (32%). In addition, we detected many unique markers in RSV-A and B that had not been seen before throughout the viral genome, along with other common markers that had been previously detected and are common in GA2 and GB5 genotypes (15, 46, 47). Since these molecular markers were unique to the Kuwaiti RSV-A and RSV-B strains, these special markers could provide a presumptive genotype classification for viruses for which sequence data are available only for genes other than the G gene.

This is the first study in Kuwait to characterize the genomes of RSV A and B to identify the circulating genotypes, subgenotypes, and even lineages of RSV genotypes, to analyze the genetic diversity of the virus, and to identify imported genetic markers associated with specific genotypes. As candidate RSV vaccines are considered for use, these data will allow a comparison of vaccine viruses with those in Kuwait. The obtained information will provide useful insights into future vaccine and therapy strategies for RSV in Kuwait.

## MATERIALS AND METHODS

### Study population and specimen collection

Retrospectively, between January 2020 and September 2022, 7,093 respiratory samples were collected from hospitalized patients with ARTIs at Mubarak Al-Kabeer Hospital. The patients had various respiratory diseases, such as bronchiolitis, pneumonia, acute respiratory distress syndrome, croup, bronchopneumonia, and acute nasopharyngitis. The collected respiratory samples were nasopharyngeal aspirates/wash, nasopharyngeal swabs, bronchoalveolar lavage, tracheal aspirates, sputum, and throat and nasal swabs. The collected samples were screened for respiratory viruses, including RSV, using real-time multiplex PCR at Mubarak Al-Kabeer Hospital’s Virology Unit. This assay can detect influenza A virus, influenza A (H1N1) virus, influenzas B virus, human rhinovirus, human coronavirus NL63 (HCoV-NL63), HCoV-229E, HCoV-OC43, HCoV-HKU1, parainfluenza virus (PIV) types 1, 2, 3, and 4, human metapneumovirus A/B, bocavirus, RSV A/B, adenovirus, enterovirus, parechovirus, mycoplasma pneumonia, and internal control, using a Fast Track Kit (Fast Track Diagnostics, Luxembourg). The samples were transferred to the Virology Unit, Faculty of Medicine, Kuwait University, and stored at −70°C until being sequenced by MinION Nanopore technology.

### Nucleic acid extraction and reverse transcriptase PCR for the G protein gene of RSV

Following the manufacturer’s instructions, the PureLink Viral RNA/DNA Mini Kit (Thermo Fisher Scientific) was used to extract viral RNA from 200 µL of the respiratory samples. RiboLocl RNase Inhibitor (Thermo Fisher Scientific) was added to the extracted samples to preserve the extracted RNA. The quantity and quality of the RNA extracts were determined using the Qubit RNA HS Assay. For samples to be sequenced, the positivity of RSV was confirmed using reverse transcriptase PCR (RT-PCR) for the G protein gene of RSV as described previously (19).

### DNase treatment

DNase I Amplification Grade (Thermo Fisher Scientific) was added to each sample as described previously (22) to remove the host DNA from the extracted samples.

### Whole-genome amplification of RSV using overlapping RT-PCR

We performed an overlapping RT-PCR strategy to amplify the whole viral genome using in-house overlapping primers targeting RSV-A and RSV-B genotypes. Nada Madi designed the primers to generate amplicons ranging in size from 337 bp to 1,129 bp that overlap by approximately 20 bp (sequences are provided upon request). A One-Step RT-PCR Kit (QIAGEN, GmbH, Hilden, Germany) synthesized and amplified the cDNA from the extracted RNA according to the manufacturer’s instructions. Briefly, 19 parallel PCR reactions were prepared to amplify the genome using 19 forward and reverse primers, 5× QIAGEN One-Step RT-PCR Buffer, dNTP mix (10 mM of each dNTP), 5× Q-solution, Hi-Di Formamide (Applied Biosystems, USA), QIAGEN One-Step RT-PCR Enzyme Mix, RNase-free water, and extracted RNA. PCR thermal profile was as follows: 37°C for 30 min, followed by 15 min at 95°C, 40 cycles of 40 sec at 94°C, 45 sec at 48.3°C, and 2 min at 72°C with a final extension of 10 min at 72°C. Different annealing temperatures were used for the amplification according to the primers’ melting temperature (Tm), ranging from 40.5°C to 48.3°C. The resulting amplicons were analyzed by 1% agarose gel electrophoresis.

### RSV whole-genome sequencing using MinION Nanopore technology

The whole genome of RSV from the clinical samples was sequenced using the Oxford Nanopore sequencing technology (Oxford Nanopore Technologies, Cambridge, United Kingdom). The amplicons were cleaned up with AMPure XP Beads (Beckman Coulter Diagnostics, California, USA), and libraries were prepared using the ligation sequencing kit (SQK-LSK109) from Oxford Nanopore Technologies (Oxford, United Kingdom) according to the manufacturer’s instructions. DNA ends were repaired and end-prepped/dA-tailed using the NEBNext Ultra End Repair/dA-Tailing Module Kits (E7546, New England BioLabs [NEB], Ipswich, MA). Next, the native barcode ligation step was performed using Native Barcoding Expansion 1–12 (EXP-NBD104) and 13–24 (EXP-NBD114) according to the manufacturer’s instructions. Then, the product was purified with AMPure XP Beads (A63880, Beckman Coulter, USA), washed with 70% ethanol, and eluted with nuclease-free water. Sequencing adapters were added to the pooled and barcoded DNA using Adapter Mix and Quick T4 DNA Ligase with Ligation Buffer (NEB) and purified with AMPure Beads. Then, libraries were quantified using the QUBIT 1× dsDNA HS Assay Kit (Invitrogen, Waltham, MA), and 15 ng of the library was loaded into Oxford Nanopore MinION SpotON Flow Cells FLO-MIN106D, R9.4.1 (Oxford Nanopore Technologies, Oxford, United Kingdom). The FastQ files generated by the Mk1C device were used for analysis.

### Data analysis

The output FASTQ files generated from the Mk1C device were base-called and de-multiplexed using Guppy version 3.1.5. The quality of reads was checked using NanoPlot (v1.41.6). The barcodes and adapters were removed from the FASTQ reads using Porechop (v0.2.4) and filtered according to their base-pair length and quality (NanoFilt -q 10 -l 300 –max length 1,600) using NanoFilt (v2.6.0). The FASTQ reads were then aligned to either of the RSV reference genomes (NC_038235.1 Human orthopneumovirus Subgroup A and NC_001781.1 Human orthopneumovirus Subgroup B). SAMTOOLS (v1.13) and BCFTOOLS (v1.5) were used to convert SAM files to sorted BAM files and to generate a FASTA consensus file. The mapping quality on both reference strains was checked using Qualimap (v.2.2.2).

Multiple sequence alignments of study consensus sequences with known RSV genotypes that were recently classified by Goya et al. (40) were carried out using MUSCLE (Multiple Sequence Comparison by Log Expectation) algorithms in MEGA (MEGA 11 v11.0.13: Molecular Evolutionary Genetics Analysis across computing platforms) (48). The published sequences of RSV-A and RSV-B reference genomes were downloaded from the National Center for Biotechnology Information (NCBI) database. The initial genotype assignment and identification were performed using the Nextstrain genetic analysis platform (49). The phylogenetic trees of nucleotide sequences for RSV-A and RSV-B subgroups were constructed with the Bayesian Information criterion using the maximum likelihood method under Tamura-Nei/JTT matrix-based models with 1,000 bootstrapping iterations with MEGA11 v11.0.13. The resulting consensus sequences of RSV-A and RSV-B strains were analyzed with sequences identified globally. From different geolocations, 960 complete genomes of RSV-A sampled between December 1977 and September 2022 and 888 complete genomes of RSV-B sampled between July 1977 and September 2022 were downloaded from GenBank of NCBI. Using the Augur pipeline (49), the global sequences were aligned to the RSV-A and RSV-B strains from Kuwait using MAFFT, and time-resolved maximum likelihood phylogenetic trees with 1,000 bootstrap replicates were constructed using IQ-Tree (50) under the GTR substitution model and visualized with Auspice (49). The Interactive Tree of Life v6 software visualized and modified the phylogenetic trees. To analyze if the identified clusters belong to distinct genotypes and calculate the genotypic and intergenotypic *P*-distance, the metric distance was computed using MEGA 11v11.0.13. The ratio between the missense substitutions per missense site (dN) and synonymous substitutions per synonymous site (dS) was calculated using the method of Nei and Gojobori with the Jukes-Cantor correction for multiple substitutions, conducted using MEGA11 v11.0.13. The dN/dS ratio is an indicator of the strength of positive (>1), negative (<1), or neutral (=1) selection pressure on the viral variant. A heatmap demonstrating the percentage of molecular markers in each of the 10 genes among RSV-A and RSV-B genotypes detected in Kuwait was performed using GraphPad Prism v9.

## Data Availability

Eighty-four RSV-A and 23 RSV-B whole-genome sequences were deposited in GenBank with the accession numbers PP135042–PP135061PP135061 and PP151342–PP151405.

## SUPPLEMENTAL MATERIAL

Supplemental material spectrum.00159-24-s0001.docx Tables S1 and S2.

ASM does not own the copyrights to Supplemental Material that may be linked to, or accessed through, an article. The authors have granted ASM a non-exclusive, world-wide license to publish the Supplemental Material files. Please contact the corresponding author directly for reuse.

## References

[1] Borchers AT, Chang C, Gershwin ME, Gershwin LJ. 2013. Respiratory syncytial virus - a comprehensive review. Clin Rev Allergy Immunol 45:331–379. doi:10.1007/s12016-013-8368-9 23575961 PMC7090643

[2] Nair H, Brooks WA, Katz M, Roca A, Berkley JA, Madhi SA, Simmerman JM, Gordon A, Sato M, Howie S, et al.. 2011. Global burden of respiratory infections due to seasonal influenza in young children: a systematic review and meta-analysis. Lancet 378:1917–1930. doi:10.1016/S0140-6736(11)61051-9 22078723

[3] Neuzil KM. 2016. Progress toward a respiratory syncytial virus vaccine. Clin Vaccine Immunol 23:186–188. doi:10.1128/CVI.00037-16 26818954 PMC4783429

[4] Han LL, Alexander JP, Anderson LJ. 1999. Respiratory syncytial virus pneumonia among the elderly: an assessment of disease burden. J Infect Dis 179:25–30. doi:10.1086/314567 9841818

[5] Falsey AR, Hennessey PA, Formica MA, Cox C, Walsh EE. 2005. Respiratory syncytial virus infection in elderly and high-risk adults. N Engl J Med 352:1749–1759. doi:10.1056/NEJMoa043951 15858184

[6] Houben ML, Bont L, Wilbrink B, Belderbos ME, Kimpen JLL, Visser GHA, Rovers MM. 2011. Clinical prediction rule for RSV bronchiolitis in healthy newborns: prognostic birth cohort study. Pediatrics 127:35–41. doi:10.1542/peds.2010-0581 21187309

[7] Hall CB, Geiman JM, Biggar R, Kotok DI, Hogan PM, Douglas GR Jr. 1976. Respiratory syncytial virus infections within families. N Engl J Med 294:414–419. doi:10.1056/NEJM197602192940803 173995

[8] Goya S, Valinotto LE, Tittarelli E, Rojo GL, Nabaes Jodar MS, Greninger AL, Zaiat JJ, Marti MA, Mistchenko AS, Viegas M. 2018. An Optimised methodology for whole genome sequencing of RNA respiratory viruses from nasopharyngeal aspirates. PLoS One 13:e0199714. doi:10.1371/journal.pone.0199714 29940028 PMC6016902

[9] Mufson MA, Orvell C, Rafnar B, Norrby E. 1985. Two distinct subtypes of human respiratory syncytial virus. J Gen Virol 66 (Pt 10):2111–2124. doi:10.1099/0022-1317-66-10-2111 2413163

[10] Cane PA, Matthews DA, Pringle CR. 1991. Identification of variable domains of the attachment (G) protein of subgroup A respiratory syncytial viruses. J Gen Virol 72 (Pt 9):2091–2096. doi:10.1099/0022-1317-72-9-2091 1895054

[11] Gaymard A, Bouscambert-Duchamp M, Pichon M, Frobert E, Vallee J, Lina B, Casalegno J-S, Morfin F. 2018. Genetic characterization of respiratory syncytial virus highlights a new BA genotype and emergence of the ON1 genotype in Lyon, France, between 2010 and 2014. J Clin Virol 102:12–18. doi:10.1016/j.jcv.2018.02.004 29471266

[12] Abou-El-Hassan H, Massaad E, Soudani N, Assaf-Casals A, Shaker R, Lteif Khoury M, Ghanem S, Karam M, Andary R, Saito R, Dbaibo G, Zaraket H. 2019. Detection of ON1 and novel genotypes of human respiratory syncytial virus and emergence of Palivizumab resistance in Lebanon. PLoS One 14:e0212687. doi:10.1371/journal.pone.0212687 30789963 PMC6383889

[13] Bose ME, He J, Shrivastava S, Nelson MI, Bera J, Halpin RA, Town CD, Lorenzi HA, Noyola DE, Falcone V, Gerna G, De Beenhouwer H, Videla C, Kok T, Venter M, Williams JV, Henrickson KJ. 2015. Sequencing and analysis of globally obtained human respiratory syncytial virus a and B genomes. PLoS One 10:e0120098. doi:10.1371/journal.pone.0120098 25793751 PMC4368745

[14] Peret TC, Golub JA, Anderson LJ, Hall CB, Schnabel KC. 1998. Circulation patterns of genetically distinct group A and B strains of human respiratory syncytial virus in a community. J Gen Virol. 79:2221–2229. doi:10.1099/0022-1317-79-9-2221 9747732

[15] Reiche J, Schweiger B. 2009. Genetic variability of group A human respiratory syncytial virus strains circulating in Germany from 1998 to 2007. J Clin Microbiol 47:1800–1810. doi:10.1128/JCM.02286-08 19386848 PMC2691087

[16] Fletcher JN, Smyth RL, Thomas HM, Ashby D, Hart CA. 1997. Respiratory syncytial virus genotypes and disease severity among children in hospital. Arch Dis Child 77:508–511. doi:10.1136/adc.77.6.508 9496185 PMC1717415

[17] Peret TCT, Hall CB, Hammond GW, Piedra PA, Storch GA, Sullender WM, Tsou C, Anderson LJ. 2000. Circulation patterns of group A and B human respiratory syncytial virus genotypes in 5 communities in North America. J Infect Dis 181:1891–1896. doi:10.1086/315508 10837167

[18] Yu J-M, Fu Y-H, Peng X-L, Zheng Y-P, He J-S. 2021. Genetic diversity and molecular evolution of human respiratory syncytial virus A and B. Sci Rep 11:12941. doi:10.1038/s41598-021-92435-1 34155268 PMC8217232

[19] Madi N, Chehadeh W, Asadzadeh M, Al-Turab M, Al-Adwani A. 2018. Analysis of genetic variability of respiratory syncytial virus groups A and B in Kuwait. Arch Virol 163:2405–2413. doi:10.1007/s00705-018-3881-z 29777370 PMC7087269

[20] Gilca R, De Serres G, Tremblay M, Vachon M-L, Leblanc E, Bergeron MG, Dery P, Boivin G. 2006. Distribution and clinical impact of human respiratory syncytial virus genotypes in hospitalized children over 2 winter seasons. J Infect Dis 193:54–58. doi:10.1086/498526 16323132

[21] Imaz MS, Sequeira MD, Videla C, Veronessi I, Cociglio R, Zerbini E, Carballal G. 2000. Clinical and epidemiologic characteristics of respiratory syncytial virus subgroups A and B infections in Santa Fe, Argentina. J Med Virol 61:76–80. doi:10.1002/(sici)1096-9071(200005)61:1<76::aid-jmv12>3.0.co;2-p 10745236

[22] Hornsleth A, Klug B, Nir M, Johansen J, Hansen KS, Christensen LS, Larsen LB. 1998. Severity of respiratory syncytial virus disease related to type and genotype of virus and to cytokine values in nasopharyngeal secretions. Pediatr Infect Dis J 17:1114–1121. doi:10.1097/00006454-199812000-00003 9877358

[23] Karron RA, Luongo C, Thumar B, Loehr KM, Englund JA, Collins PL, Buchholz UJ. 2015. A gene deletion that up-regulates viral gene expression yields an attenuated RSV vaccine with improved antibody responses in children. Sci Transl Med 7:312ra175. doi:10.1126/scitranslmed.aac8463 PMC6342448 26537255

[24] Sullender WM. 2000. Respiratory syncytial virus genetic and Antigenic diversity. Clin Microbiol Rev 13:1–15, doi:10.1128/CMR.13.1.1 10627488 PMC88930

[25] Trento A, Ábrego L, Rodriguez-Fernandez R, González-Sánchez MI, González-Martínez F, Delfraro A, Pascale JM, Arbiza J, Melero JA. 2015. Conservation of G-protein epitopes in respiratory syncytial virus (group A) despite broad genetic diversity: is antibody selection involved in virus evolution? J Virol 89:7776–7785. doi:10.1128/JVI.00467-15 25995258 PMC4505632

[26] Fan R, Fan C, Zhang J, Wen B, Lei Y, Liu C, Chen L, Liu W, Wang C, Qu X. 2017. Respiratory syncytial virus subtype ON1/NA1/BA9 predominates in hospitalized children with lower respiratory tract infections. J Med Virol 89:213–221. doi:10.1002/jmv.24619 27358012 PMC7166484

[27] Midulla F, Di Mattia G, Nenna R, Scagnolari C, Viscido A, Oliveto G, Petrarca L, Frassanito A, Arima S, Antonelli G, Pierangeli A. 2020. Novel variants of respiratory syncytial virus A On1 associated with increased clinical severity of bronchiolitis. J Infect Dis 222:102–110. doi:10.1093/infdis/jiaa059 32031626

[28] Cui G, Zhu R, Qian Y, Deng J, Zhao L, Sun Y, Wang F. 2013. Genetic variation in attachment glycoprotein genes of human respiratory syncytial virus subgroups A and B in children in recent five consecutive years. PLoS One 8:e75020. doi:10.1371/journal.pone.0075020 24069376 PMC3775769

[29] Muñoz-Escalante JC, Comas-García A, Bernal-Silva S, Noyola DE. 2021. Respiratory syncytial virus B sequence analysis reveals a novel early genotype. Sci Rep 11:3452. doi:10.1038/s41598-021-83079-2 33568737 PMC7876121

[30] Madi N, Chehadeh W, Asadzadeh M, Al-Turab M, Al-Adwani A. 2018. Analysis of genetic variability of respiratory syncytial virus groups A and B in Kuwait. Arch Virol 163:2405–2413. doi:10.1007/s00705-018-3881-z 29777370 PMC7087269

[31] Panatto D, Domnich A, Lai PL, Ogliastro M, Bruzzone B, Galli C, Stefanelli F, Pariani E, Orsi A, Icardi G. 2023. Epidemiology and molecular characteristics of respiratory syncytial virus (RSV) among Italian community-dwelling adults, 2021/22 season. BMC Infect Dis 23:134. doi:10.1186/s12879-023-08100-7 36882698 PMC9990006

[32] Bénet T, Sánchez Picot V, Messaoudi M, Chou M, Eap T, Wang J, Shen K, Pape J-W, Rouzier V, Awasthi S, et al.. 2017. Microorganisms associated with pneumonia in children <5 years of age in developing and emerging countries: the GABRIEL pneumonia multicenter, prospective, case-control study. Clin Infect Dis 65:604–612. doi:10.1093/cid/cix378 28605562 PMC7108107

[33] Luo Q, Li M, Li A, Gong C, Dong M, Huang Q, Luo M, Zhang H, Huang F. 2022. Genetic diversity and epidemiological features of respiratory syncytial virus, Beijing, 2015–2019: a multicenter and all-age groups study. J Infect 85:75–85. doi:10.1016/j.jinf.2022.04.046 35533834

[34] Fagbo SF, Garbati MA, Hasan R, AlShahrani D, Al-Shehri M, AlFawaz T, Hakawi A, Wani TA, Skakni L. 2017. Acute viral respiratory infections among children in MERS‐endemic Riyadh, Saudi Arabia, 2012–2013. J Med Virol 89:195–201. doi:10.1002/jmv.24632 27430485 PMC7166860

[35] Zhao X, Wang C, Jiang H, Zhang H, Fang F, Chen M, Yuan Z, Teng Z, Liu J, Zhang X. 2022. Analysis of circulating respiratory syncytial virus A strains in Shanghai, China identified a new and increasingly prevalent lineage within the dominant ON1 genotype. Front Microbiol 13. doi:10.3389/fmicb.2022.966235 PMC9403419 36033866

[36] Yu J, Liu C, Xiao Y, Xiang Z, Zhou H, Chen L, Shen K, Xie Z, Ren L, Wang J. 2019. Respiratory syncytial virus seasonality, Beijing, China, 2007–2015. Emerg Infect Dis 25:1127–1135. doi:10.3201/eid2506.180532 31107230 PMC6537707

[37] Obando-Pacheco P, Justicia-Grande AJ, Rivero-Calle I, Rodríguez-Tenreiro C, Sly P, Ramilo O, Mejías A, Baraldi E, Papadopoulos NG, Nair H, Nunes MC, Kragten-Tabatabaie L, Heikkinen T, Greenough A, Stein RT, Manzoni P, Bont L, Martinón-Torres F. 2018. Respiratory syncytial virus Seasonality: a global overview. J Infect Dis 217:1356–1364. doi:10.1093/infdis/jiy056 29390105

[38] Zhang Z-Y, Du L-N, Chen X, Zhao Y, Liu E-M, Yang X-Q, Zhao X-D. 2010. Genetic variability of respiratory syncytial viruses (RSV) prevalent in southwestern China from 2006 to 2009: emergence of subgroup B and A RSV as dominant strains. J Clin Microbiol 48:1201–1207. doi:10.1128/JCM.02258-09 20147636 PMC2849604

[39] Parveen S, Sullender WM, Fowler K, Lefkowitz EJ, Kapoor SK, Broor S. 2006 a. Genetic variability in the G protein gene of group A and B respiratory syncytial viruses from India. J Clin Microbiol 44:3055–3064. doi:10.1128/JCM.00187-06 16954227 PMC1594720

[40] Goya S, Lucion MF, Shilts MH, Juárez MDV, Gentile A, Mistchenko AS, Viegas M, Das SR. 2023. Evolutionary dynamics of respiratory syncytial virus in Buenos Aires: viral diversity, migration, and subgroup replacement. Virus Evol 9:vead006. doi:10.1093/ve/vead006 36880065 PMC9985318

[41] Agrawal AS, Sarkar M, Ghosh S, Chawla-Sarkar M, Chakraborty N, Basak M, Naik TN. 2009. Prevalence of respiratory syncytial virus group B genotype BA-IV strains among children with acute respiratory tract infection in Kolkata, Eastern India. J Clin Virol 45:358–361. doi:10.1016/j.jcv.2009.05.013 19570709

[42] Bandla SS, Devadiga S, Bhatt R, Dsa OC, Govindakarnavar A. 2021. Molecular epidemiology of respiratory syncytial virus among children and adults in India 2016 to 2018. Virus Genes 57:489–501. doi:10.1007/s11262-021-01859-4 34524602 PMC8440155

[43] Goya S, Galiano M, Nauwelaers I, Trento A, Openshaw PJ, Mistchenko AS, Zambon M, Viegas M. 2020. Toward unified molecular surveillance of RSV: a proposal for genotype definition. Influenza Other Respir Viruses 14:274–285. doi:10.1111/irv.12715 32022426 PMC7182609

[44] Scudero OB, Santiago VF, Palmisano G, Simabuco FM, Ventura AM. 2023. The respiratory syncytial virus M2-2 protein is targeted for proteasome degradation and inhibits translation and stress granules assembly. PLoS One 18:e0289100. doi:10.1371/journal.pone.0289100 37490507 PMC10368288

[45] Perez M, García-Barreno B, Melero JA, Carrasco L, Guinea R. 1997. Membrane permeability changes induced in Escherichia coli by the SH protein of human respiratory syncytial virus. Virology 235:342–351. doi:10.1006/viro.1997.8696 9281514

[46] Arnott A, Vong S, Mardy S, Chu S, Naughtin M, Sovann L, Buecher C, Beauté J, Rith S, Borand L, Asgari N, Frutos R, Guillard B, Touch S, Deubel V, Buchy P. 2011. A study of the genetic variability of human respiratory syncytial virus (HRSV) in cambodia reveals the existence of a new HRSV group B genotype. J Clin Microbiol 49:3504–3513. doi:10.1128/JCM.01131-11 21865418 PMC3187327

[47] Suwa R, Kume Y, Kawase M, Chishiki M, Ono T, Norito S, Sato K, Okamoto M, Kumaki S, Nagai Y, Hosoya M, Takeda M, Nishimura H, Hashimoto K, Shirato K. 2022. Practical validation of United States centers for disease control and prevention assays for the detection of human respiratory syncytial virus in pediatric inpatients in Japan. Pathogens 11:754. doi:10.3390/pathogens11070754 35889999 PMC9319774

[48] Tamura K, Stecher G, Kumar S. 2021. Mega11: Molecular evolutionary Genetics analysis version 11. Mol Biol Evol 38:3022–3027. doi:10.1093/molbev/msab120 33892491 PMC8233496

[49] Hadfield J, Megill C, Bell SM, Huddleston J, Potter B, Callender C, Sagulenko P, Bedford T, Neher RA. 2018. Nextstrain: real-time tracking of pathogen evolution. Bioinformatics 34:4121–4123. doi:10.1093/bioinformatics/bty407 29790939 PMC6247931

[50] Nguyen L-T, Schmidt HA, von Haeseler A, Minh BQ. 2015. IQ-TREE: A fast and effective stochastic algorithm for estimating maximum-likelihood Phylogenies. Mol Biol Evol 32:268–274. doi:10.1093/molbev/msu300 25371430 PMC4271533

