# Supplementary material for: Genomic characterization of circulating human respiratory syncytial viruses A and B in Kuwait using whole-genome sequencing
Source: Microbiol Spectr. 2024 May 29;12(7):e00159-24. doi: 10.1128/spectrum.00159-24 (PMC11218466; doi:10.1128/spectrum.00159-24)
Supplement: Supplemental material — Tables S1 and S2. [file spectrum.00159-24-s0001.docx]

**Supplementary**

Table S1. RSV-A genotype amino acid markers.

| **Protein** | **Amino acid (AA) substitution** | **No. of AA changes** | **Percentage of changes** |
| --- | --- | --- | --- |
| **NS1** | H36Q | 61* | 94 |
| **NS2** | **T26I** | 64 | 100 |
|  | R38K | 64 | 100 |
| **N** | R84K | 62 | 97 |
|  | H216Y | 60 | 94 |
| **P** | T66I | 63 | 98 |
|  | **N72T** | 64 | 100 |
|  | A73V | 62 | 95 |
|  | **I143M**  **I143T** | 37  22 | 92 |
| **M** | L31V | 64 | 100 |
| **SH** | **T38I** | 64 | 100 |
| **G** | **K15R** | 64 | 100 |
|  | **G29C** | 64 | 100 |
|  | **T57A** | 64 | 100 |
|  | S81N | 64 | 100 |
|  | D94N | 64 | 100 |
|  | E106G | 64 | 100 |
|  | I107T | 64 | 100 |
|  | T111S | 64 | 100 |
|  | G121S | 64 | 100 |
|  | V122A | 64 | 100 |
|  | K123E | 64 | 100 |
|  | **N125T** | 64 | 100 |
|  | L126P | 64 | 100 |
|  | **P128S** | 64 | 100 |
|  | T133I | 64 | 100 |
|  | T141I | 64 | 100 |
|  | Q142L | 64 | 100 |
|  | P156Q | 64 | 100 |
|  | **F208L** | 64 | 100 |
|  | L215P | 64 | 100 |
|  | L253P | 61 | 95 |
|  | E232G | 48 | 75 |
|  | E233K | 51 | 80 |
|  | T244I | 64 | 100 |
|  | N250S | 64 | 100 |
|  | T253K | 53 | 83 |
|  | **K257E** | 64 | 100 |
|  | L258H  **L258Q** | 22  36 | 91 |
|  | **M262E**  **M262K** | 38  11 | 77 |
|  | **F265L** | 55 | 86 |
|  | S269T | 52 | 81 |
|  | N273Y | 51 | 80 |
|  | L274P | 60 | 94 |
|  | **S280Y** | 50 | 78 |
|  | **H285Y** | 63 | 98 |
|  | **T296P** | 61 | 95 |
| **F** | A8T | 62 | 97 |
|  | F20L | 56 | 88 |
|  | **N80K** | 64 | 100 |
|  | **T101P** | 64 | 100 |
|  | **N163S** | 64 | 100 |
|  | K124N | 62 | 97 |
|  | R213S | 64 | 100 |
|  | N276S | 60 | 94 |
|  | V384I | 62 | 97 |
|  | **H515N** | 64 | 100 |
|  | S540A | 60 | 94 |
| **M2-1** | L120P | 62 | 97 |
|  | I125V | 62 | 97 |
|  | L179S | 64 | 100 |
| **M2-2** | M1T | 52 | 82 |
|  | T18N | 62 | 97 |
|  | R25N | 62 | 97 |
|  | F33Q | 64 | 100 |
|  | F39I | 62 | 97 |
|  | P44Q | 61 | 95 |
|  | M48I | 61 | 95 |
|  | S50P | 61 | 95 |
|  | N52D | 59 | 92 |
|  | I54P | 61 | 95 |
|  | T68A | 61 | 95 |
|  | I69T | 61 | 95 |
|  | **V78I** | 63 | 98 |
|  | **I79A** | 58 | 91 |
|  | E80D | 62 | 97 |
| **L** | N6S | 61 | 95 |
|  | M59I | 60 | 94 |
|  | L81 | 60 | 94 |
|  | **L100S** | 64 | 100 |
|  | I103T | 61 | 95 |
|  | A104T | 62 | 97 |
|  | S162S | 62 | 97 |
|  | H177Q | 62 | 97 |
|  | N216S | 59 | 92 |
|  | N224S | 60 | 94 |
|  | Q237H | 59 | 92 |
|  | **F424L** | 62 | 97 |
|  | **N547S** | 59 | 92 |
|  | E575D | 64 | 100 |
|  | I598H | 60 | 94 |
|  | R754K | 61 | 95 |
|  | H1471N | 64 | 100 |
|  | **K1590R** | 61 | 95 |
|  | H1657Y | 61 | 95 |
|  | N1700S | 60 | 94 |
|  | A1718T | 60 | 94 |
|  | I1721V | 59 | 92 |
|  | D1725G | 60 | 94 |
|  | N1730S | 57 | 89 |
|  | L1745S | 59 | 92 |
|  | **V1752I** | 64 | 100 |
|  | **A1755T** | 59 | 92 |
|  | K1764R | 64 | 100 |
|  | R1778K | 61 | 95 |
|  | **I2016V** | 61 | 95 |
|  | Y2135H | 62 | 97 |

*Number of sequences that present the specific amino acid

changes in the genotype that is considered as molecular marker.

AA substitutions in bold are unique molecular markers.

Supplementary Table 2. RSV-B genotype amino acid markers.

| **Protein** | **Amino acid (AA) substitution** | **No. of AA changes** | **Percentage of changes** |
| --- | --- | --- | --- |
| **NS1** | A45T | 20* | 100 |
|  | M105I | 20 | 100 |
|  | N124D | 20 | 100 |
|  | N138H | 20 | 100 |
| **NS2** | - | - | - |
| **N** | H216Y | 20 | 100 |
|  | A372T | 20 | 100 |
|  | **N380H** | 19 | 95 |
| **P** | **P215S** | 18 | 90 |
| **M** | I89T | 18 | 90 |
|  | **P166S** | 18 | 90 |
| **SH** | - | - | - |
| **G** | P95S | 20 | 100 |
|  | S101P | 20 | 100 |
|  | T107A | 20 | 100 |
|  | S109P | 20 | 100 |
|  | T118I | 20 | 100 |
|  | R136T | 20 | 100 |
|  | T138S | 20 | 100 |
|  | S140P | 20 | 100 |
|  | T143N | 20 | 100 |
|  | L152P | 20 | 100 |
|  | **I200T** | 17 | 85 |
|  | **T229I** | 17 | 85 |
|  | **L237P** | 17 | 85 |
|  | **T238K** | 19 | 90 |
|  | S242P | 17 | 85 |
|  | L257P | 20 | 100 |
|  | E258K | 20 | 100 |
|  | I261T | 19 | 95 |
|  | T270I | 20 | 100 |
|  | T292I | 19 | 95 |
|  | **Q293L^#^** | 17 | 85 |
|  | N294k | 18 | 90 |
|  | H298Y | 20 | 100 |
| **F** | **L8S** | 20 | 100 |
|  | F45L | 20 | 100 |
|  | A103V | 20 | 100 |
|  | I206M | 20 | 100 |
|  | Q209R | 20 | 100 |
|  | **N234T** | 20 | 100 |
|  | T529A | 16 | 80 |
| **M2-1** | **N142S** | 20 | 100 |
|  | **I172T** | 20 | 100 |
|  | V181I | 20 | 100 |
|  | **N182S** | 20 | 100 |
| **M2-2** | **F23L** | 20 | 100 |
|  | **G45R** | 20 | 100 |
|  | A49V | 20 | 100 |
|  | **N85S** | 20 | 100 |
| **L** | N8S | 20 | 100 |
|  | **L58I** | 20 | 100 |
|  | **S166N** | 20 | 100 |
|  | H177Y | 20 | 100 |
|  | T184N | 20 | 100 |
|  | N374D | 20 | 100 |
|  | I715V | 20 | 100 |
|  | M973T | 20 | 100 |
|  | S1250G | 20 | 100 |
|  | **N1471H** | 20 | 100 |
|  | V1479A` | 20 | 100 |
|  | K1547R | 20 | 100 |
|  | T1712A | 20 | 100 |
|  | I1716V | 20 | 100 |
|  | S1726R | 20 | 100 |
|  | N1736S | 20 | 100 |
|  | **I1740M** | 20 | 100 |
|  | **M1742T** | 20 | 100 |
|  | K1764R | 20 | 100 |
|  | **N1773I** | 20 | 100 |
|  | N1787E | 20 | 100 |
|  | **I1794T** | 20 | 100 |
|  | **F2030L** | 20 | 100 |
|  | T2042I | 20 | 100 |
|  | K2065N | 20 | 100 |

*Number of sequences that present the specific amino acid changes in the genotype that is considered a molecular marker.

^#^ Stop codon

AA substitutions in bold are unique molecular markers
